# Prediction of Rat Behavior Outcomes in Memory Tasks Using Functional Connections among Neurons

**DOI:** 10.1371/journal.pone.0074298

**Published:** 2013-09-30

**Authors:** Hu Lu, Shengtao Yang, Longnian Lin, Baoming Li, Hui Wei

**Affiliations:** 1 School of Computer Science, Fudan University, Shanghai, China; 2 Institute of Neurobiology and State Key Laboratory of Medical Neurobiology, Fudan University, Shanghai, China; 3 Key Laboratories of MOE and STCSM, Shanghai Institute of Brain Functional Genomics, East China Normal University, Shanghai, China; University of Michigan, United States of America

## Abstract

**Background:**

Analyzing the neuronal organizational structures and studying the changes in the behavior of the organism is key to understanding cognitive functions of the brain. Although some studies have indicated that spatiotemporal firing patterns of neuronal populations have a certain relationship with the behavioral responses, the issues of whether there are any relationships between the functional networks comprised of these cortical neurons and behavioral tasks and whether it is possible to take advantage of these networks to predict correct and incorrect outcomes of single trials of animals are still unresolved.

**Methodology/Principal Findings:**

This paper presents a new method of analyzing the structures of whole-recorded neuronal functional networks (WNFNs) and local neuronal circuit groups (LNCGs). The activity of these neurons was recorded in several rats. The rats performed two different behavioral tasks, the Y-maze task and the U-maze task. Using the results of the assessment of the WNFNs and LNCGs, this paper describes a realization procedure for predicting the behavioral outcomes of single trials. The methodology consists of four main parts: construction of WNFNs from recorded neuronal spike trains, partitioning the WNFNs into the optimal LNCGs using social community analysis, unsupervised clustering of all trials from each dataset into two different clusters, and predicting the behavioral outcomes of single trials. The results show that WNFNs and LNCGs correlate with the behavior of the animal. The U-maze datasets show higher accuracy for unsupervised clustering results than those from the Y-maze task, and these datasets can be used to predict behavioral responses effectively.

**Conclusions/Significance:**

The results of the present study suggest that a methodology proposed in this paper is suitable for analysis of the characteristics of neuronal functional networks and the prediction of rat behavior. These types of structures in cortical ensemble activity may be critical to information representation during the execution of behavior.

## Introduction

The cerebral cortex is a dynamic network composed of a large number of neurons joined to each other by synaptic connections. Early theories maintained that brain information was represented by the firing rates of single neurons. The new theories of brain functions propose that the information transmission and processing of the central nervous system are represented by the spatiotemporal firing patterns of large neuronal populations. Spatiotemporal patterns refer to a series of regular and ordered interval spikes among neurons. In recent years, the discovery and analysis of the spatiotemporal firing patterns have been the focus of neuroscience research [1]–[4].

One of the questions addressed in this study is how animals control the different behaviors that they perform under different stimulation conditions. This is a crucial issue in developing brain-computer interface [5]. Studies have shown that a large number of neuronal activities represent behavioral information in corresponding regions of the cerebral cortex, with different firing patterns. Studies have suggested that spatiotemporal firing patterns may be related to these behaviors. Some studies have taken advantage of these cortical activities to predict different behavioral outcomes, and control hand trajectory in brain-machine interface [6]–[11].

With the development of multi-electrode recording techniques, the activity of dozens of neurons can be recorded at one time [12]–[14]. The development of new methods of analyzing data from multiple neural spike trains has recently become a challenge in computational neuroscience [15]. In addition, the analysis of functional connections between neurons may be more meaningful than spatiotemporal firing patterns. In this way, research into the brain functional network, also known as human connectome, is opening up [16], [17]. Over the past few decades, graph theoretical analysis has been widely used for the analysis of brain functional networks [18], [19]. The brain functional network can be represented as a graph made up of statistical correlations between brain signals (e.g., fMRI, EEG, and MEG). Nodes can be neurons or cortical areas, and the networks can be weighted or unweighted, directed or undirected. The traditional brain functional networks are mainly used to analyze the characteristics of small-world network structures. They are highly efficient and highly cost-effective [20], [21]. Small-worldness can be used to compare brain's differences and as a diagnostic criterion for Alzheimer's disease (AD) [22]. They can also be used to analyze sub-modules and determine whether there is a hub node in the brain [23]. Recently brain functional network studies have focused fMRI data of the human brain [24], [25]. Other studies have extracted small-world network topology from neuronal functional networks [26]. Currently, there is no study that includes analysis of the other characteristics of neuronal functional networks, such as module structure.

The spike activities of these neuronal populations can be used to construct the WNFNs, and these networks can be divided into sub-modules. It is not known whether these network features can be used to represent behavior information. The issue of whether the functional network measurements can be used to make predictions of behavioral outcome of single trials is also unresolved. There are no relevant studies to illustrate these key issues. To solve this problem, we present a new method for investigation of the relationship between functional networks and rat behavioral choice based on the WNFNs and LNCGs obtained from multi-electrode recordings, and of using functional networks of single trials to predict the correct and incorrect outcomes of these trials. We recorded the spike activities of neurons in several rats in three areas of the cerebral cortex. The rats were trained to perform two different tasks (Y-maze and U-maze). In each behavioral task, each rat must select one of two directions in which to move. In the Y-maze task, the rat was required to choose to move left choice (L-choice) the first time and right choice the second time (R-choice). In the U-maze task, their two trial types are clockwise and counterclockwise direction. Each type is considered a trial. The spike trains between the start and end of this trial were extracted and used to construct the neuronal functional network. Each dataset of each task contained several trials. This method consists of four parts: construction of WNFNs from recorded neuronal spike trains, partitioning of these WNFNs into the optimal LNCGs using social community analysis method, unsupervised clustering all trials in each task into two different clusters, and predicting the behavioral outcomes of single trials. The results show that this method can be used to analyze the characteristics of the network structure and to predict single trial outcomes for behavior tasks. To the best of our knowledge, this is the first such study of functional brain networks and behavior prediction.

## Materials and Methods

**Figure 1:**
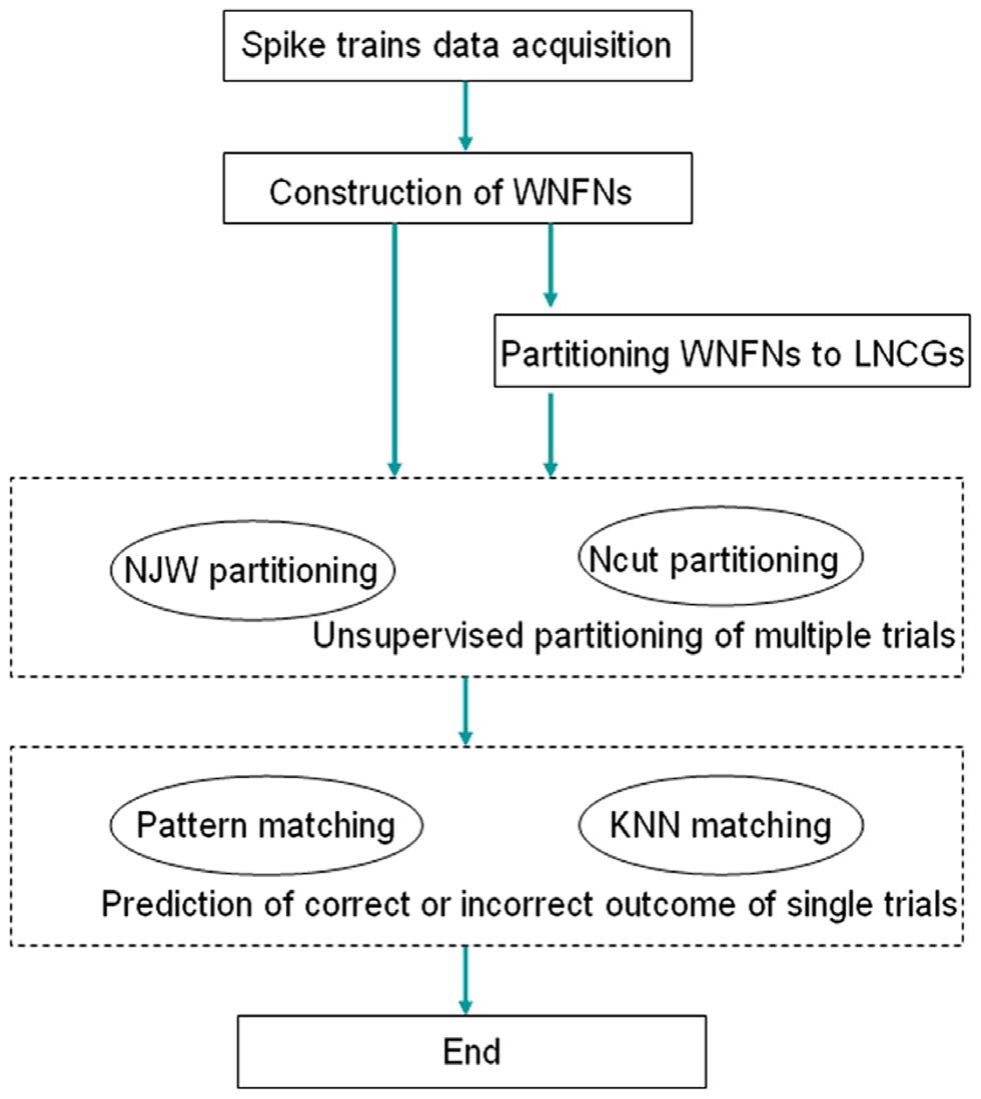
Flowchart of the method proposed in this paper.

Figure 1 shows a flowchart of the method proposed in this paper. First, we divided a recorded spike train's dataset into multiple trials in accordance with the start and end times of each trial. Then, we calculated the functional correlations of pair of neuronal spike trains and constructed WNFNs for each trial. For a dataset containing n trials, we constructed n networks. Second, a method of social network community structure analysis was adapted for partitioning WNFNs into the corresponding LNCGs. Third, we used two different unsupervised clustering methods to spectral cluster WNFNs and LNCGs for all trials of each dataset into two classes, and calculated the correctness of this partitioning. Finally, we used two different leave one cross-validation methods to predict whether the outcomes of single trials would be correct or incorrect, and to calculate the correctness of the prediction.

### Spike train data acquisition

All rat work described in this study have been conducted according to Animals Act, 2006 (China) and approved by the Institutional Animal Care and Use Committee of the East China Normal University and Institutes of Brain Science of Fudan University.

Seven male Sprague-Dawley rats (8–10 weeks old, 250∼350 g of body weight) were trained. We recorded the neuronal firing activities from seven adult male rats. The rats were deprived of drinking water for one day. The rats had to perform the two different behavioral tasks. Multi-electrode arrays were inserted into different cortical areas (the prefrontal cortex or anterior cingulate cortex for the Y-maze task, hippocampus for the U-maze task). Because the tasks were relatively simple, the rats could learn the behavior easily and became fully trained after several days. None of the rats made many incorrect choices, which less than 10%, during the remainder of the experiment. We then began to record the activity of neurons.

**Figure 2:**
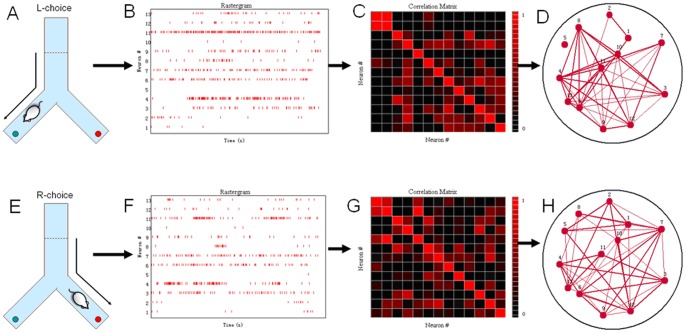
Method overview of construction of WNFNs of two trials of a Y-maze task dataset. (A) A rat performed the L-choice trial. (B) Raster plot of thirteen neurons recorded in this trial. (C) Pearson correlation matrix between pairs of neurons. (D) Neuronal functional network of these neurons. (E) – (H) Illustration of the procedure for construction of neuronal functional networks for the R-choice trial.

The Y-maze training box is a Y-shaped box. It has three arms at angles of 120 degrees. The choice arm was 71 cm and the two award arms were 42 cm. The height of the maze was 20 cm. In the Y-maze task, the rats ran from the waiting area to left arm or right arm of the box and received a water reward, indicating the completion of the trial. The rats were supposed to choose a different arm in each trial. For example, if the first correct choice was the right arm of the Y-maze (R-choice), then in the next trial, the correct choice would be the left arm of the Y-maze (L-choice). The rats need to go back to the end of the choice arm to start the next trial after the reward was consumed. The rat was to make an R-choice and then an L-choice in alternation for the duration of the test (Figure 2).

**Figure 3:**
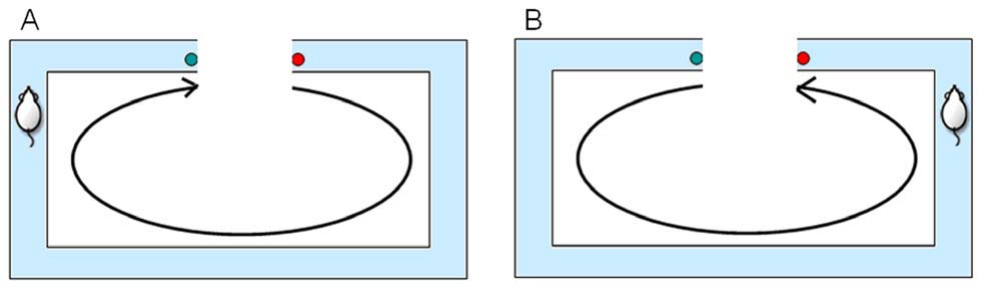
Two different trial types in the U-maze task. The rat ran back and forth along a rectangular track and received the water rewards at both ends of the track (the green and red points). (A) A rat running from the start point to the end point of the track in a clockwise direction, indicating the completion of a trial. (B) A rat finishing a different trial, having moved in a counterclockwise direction.

The U-maze training box is a rectangular track (100×80 cm). The rat had to run back and forth along the track. The rat ran from the start point (red dot) to the end point (green dot) in the correct direction (clockwise or the counterclockwise). This was considered one trial. The rat received the water reward at both ends of the track. The experimental platform is shown in Figure 3.

The activities of neuronal populations were recorded using multi-electrode arrays (cerebus-128 of blackrock microsystems technology company, USA or Plexon Inc., TX, USA). The arrays were inserted into the cerebral cortex vertically. In the Y-maze task, a microdrive array was implanted in the PFC (2.5∼4.5 mm anterior to bregma and 0.3∼0.8 mm lateral to the bregma, 2 mm ventral from brain surface). After surgery, the rats were allowed to recover for several days and then signals were recorded. In the U-maze task, a 64-channel (a bundle of 16 tetrodes) electrode was positioned above the bilateral dorsal hippocampi (3.0 mm lateral to the bregma and 4.5 mm posterior to the bregma). The electrodes were then reached the hippocampal CA1 region for recording. Offline sorter software (Plexon Inc., TX, USA) was used to cluster the neuronal firing waveforms of each electrode and distinguish the individual neuronal action potentials. For each neuron, the spikes constituted spike trains of that single neuron.

All experiments were performed in accordance with animal protocols approved by the United States National Institutes of Health (NIH). All experimental process and recording process was controlled by computer. The experimental process was monitored by a video system.

In this study, a total of ten spike trains datasets were used. Five datasets were selected from the Y-maze task and five others from the U-maze task. The number of neurons, site of the cortical recording, and number of trials per each dataset are displayed in Table 1.

**Table 1:** Datasets description of two behavioral tasks.

Dataset	Task	Place of recording	Number of neurons	Number of trials
Data 1	Y-maze	PFC	13	34
Data 2	Y-maze	PFC	10	106
Data 3	Y-maze	PFC	10	144
Data 4	Y-maze	ACC	15	62
Data 5	Y-maze	ACC	16	30
Data 6	U-maze	Hippocampus	9	80
Data 7	U-maze	Hippocampus	9	178
Data 8	U-maze	Hippocampus	17	142
Data 9	U-maze	Hippocampus	10	72
Data 10	U-maze	Hippocampus	13	90

In the above datasets, Data 1–5 were recorded from five different rats at different times. Data 6 was recorded from another rat, and Data 7–10 were recorded from the same rat at different times. Due to the different times of the recordings, the number of neurons and trials in each dataset are different.

### Construction of neuronal functional networks

Each trial was composed of neuronal spike trains. Multiple neuronal spike trains per trial were built into a neuronal functional network. Several networks can be constructed for each dataset. Each neuronal spike trains were divided into short, non-overlapping time windows (referred to as bins) and the number of spikes in each time window was calculated. In this way, the original spike trains were represented with multi-dimensional vectors. Then, the correlations between pairs of neurons were calculated using Pearson correlation coefficient [27]–[29]. The Pearson correlation coefficient is a simple linear method, but it requires bin spike activity. Binless methods have also been used [30]. In recent years, the nonlinear methods have been used to measure synchronization in the brain [31]. For the sake of generality, we have avoided creating a fully weighted network and reduced its edges. We only focused on positive Pearson correlation coefficients, although negative coefficients were also detected.



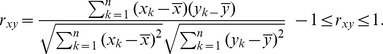
(1)Here, *x_k_* and *y_k_* denote the number of spikes of the *x*th and *y*th neurons in the *k*th bin, which have means of 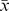 and 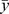, respectively. A weight matrix R is created. 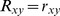, if 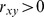. The resulting network can be represented mathematically using an adjacency matrix R. The value of the correlation coefficient *r_xy_* is between 0 and 1. A value of 1 indicates a strongest functional connection between a pair of neurons, and 0 indicates no connection.

For simple studies, traditional methods of analyzing brain functional networks usually involve converting the network into a binary network, formed by thresholding matrix R by a threshold T. All values of the correlation matrix >T were then set to 1, and others were set to 0 [32]. The threshold T is difficult to choose. The resulting network may not be in accordance with the actual network. In order to improve analysis, we directly analyzed the weighted functional connection matrix R. The weighted matrix may be of more practical significance than a binary matrix. In this way, the importance of connecting edges is represented by thickness of the lines (the thicker of lines, the stronger correlation).

### Partitioning WNFNs into LNCGs

The networks shown in Figure 2 were constructed using whole-recorded neurons in a behavior task. We refer to these as whole-recorded neuronal functional networks (WNFNs). In order to analyze the impact of local neuronal functional network on the animal behavioral types, we used the community structure analysis to divide the WNFNs into several sub-modules, which are here called local neuronal circuit groups (LNCGs). In social networks, a community structure is defined as a group of nodes with a high density of intra-group connections and a low density of inter-group connections.

To assess the best division of the WNFNs into LNCGs, we used a widely used modularity function Q, which was proposed by Newman [33], [34]. The modularity Q for a given partition of a weighted network is defined as follows:
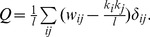
(2)Here, *l* is the total weight of all connections in the network, *w_ij_*  =  *R_ij_*, and *k_i_* and *k_j_* are the degrees of each node. *δ_ij_* is the Kronecker delta symbol and 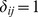, if nodes *i* and *j* are in the same community and 0 otherwise.

Given a partitioning, Q represents the difference between the actual connections and randomly connected networks with the same partition. Therefore, if a brain network is not a random network, we can divide the network into different sub-modules by maximizing the value of Q. The process consists of two steps. The first step is to obtain two partitioning results. We used a hierarchical clustering algorithm to obtain a division of neuronal functional network using the functions “linkage” and “dendrogram” in the MATLAB Toolbox. Of course, other spectral clustering methods can also be used [35]. In the second step, we calculated the corresponding Q value. We repeated these two steps using increasing number of communities and until the maximum value of Q was reached. Recently, community structure analysis methods based on modularity function Q have been used in fMRI networks [36]–[38].

**Figure 4:**
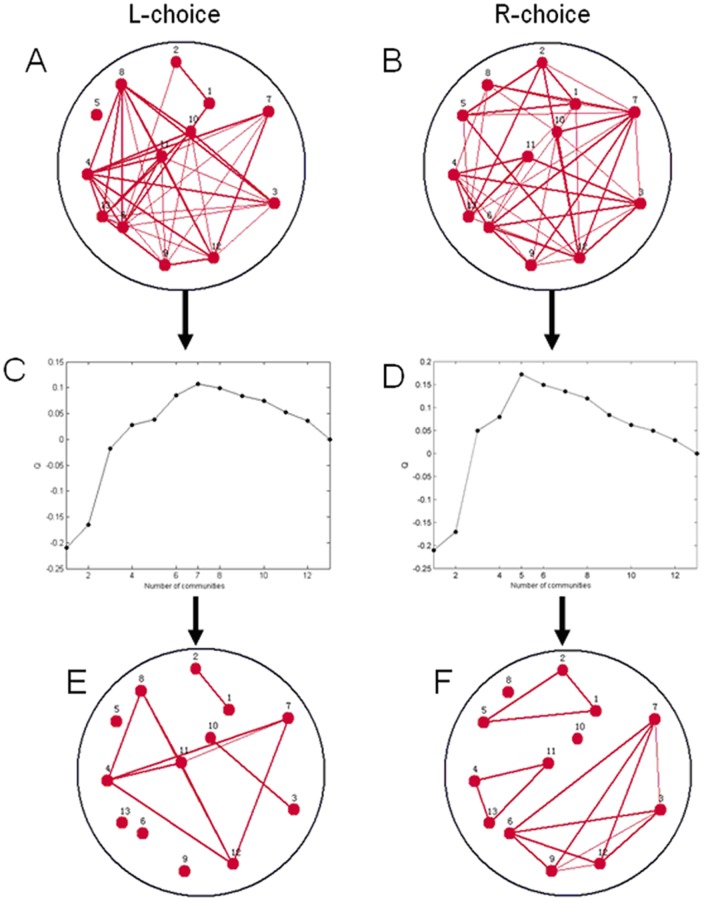
Description of procedure for dividing two networks into the best community structures. (A) WNFN in the L-choice task (Figure 2D). (B) WNFN in the R-choice task (Figure 2H). (C) Different Q values corresponding to the different number of communities in the L-choice task. (D) Different Q values corresponding to the different number of communities in the R-choice task. (E) LNCGs based on the maximum Q value in the L-choice task. (F) LNCGs based on the maximum Q value in the R-choice task.

As shown in Figure 4, a neuronal functional network of the L-choice task can be divided into seven optimal sub-modules, while the R-choice network has five sub-modules. The numbers of sub-modules of two networks are different. Nodes lacking connections become outlier nodes.

### Unsupervised clustering multiple trials

Each dataset consisted of multiple trials of each behavioral task. These can be represented by multiple neuronal functional networks. Whether these networks have a relationship with the behavior choices of the rats remains unclear. This was because each behavioral task only contained two different behavioral trial types. In this way, this is a typical two-way partitioning problem. Assuming that we do not know the rat's choice of action in advance, there is some question regarding whether we can divide all trials in each dataset into two groups corresponding to the two types of behavioral choice processes. Here, we used unsupervised clustering methods to divide the WNFNs and LNCGs of a dataset into two groups, and to calculate the accuracy of the clustering results.

For a dataset consisting of k trials on N neurons, 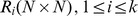 represents the connection matrix of the *i*th trial. The functional similarity between the *i*th trial and *j*th trial is defined as Gaussian kernel function, which is commonly used in graph-based approaches.
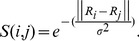
(3)Here, 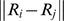 is given by 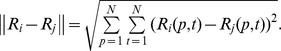


Because neuronal functional networks are represented by a graph, graph theory is a superb tool for partitioning networks. To compare the experimental results of the different methods, we realized two state-of-the-art graph-based partitioning approaches, the Ng-Jordan-Weiss (NJW), and the normalized cut (Ncut) spectral clustering algorithm [39], [40]. NJW is a classic spectral clustering algorithm. The main idea underlying this method is to convert the weight matrix to Laplace matrix.
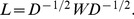
(4)Here, D is a degree matrix and W is the similarity matrix, W = S. The W matrix can be projected onto a low-dimension sub-space by finding the k largest feature vectors V (v_1_, v_2_, v_k_) and then using a traditional K-means algorithm to divide the new vectors V into k clusters. The Ncut algorithm is very relevant to the spectral clustering algorithm. It actually used the second small eigenvectors of Laplace matrix (the Fiedler vector). A two-way normalized cut is defined as follows:
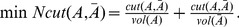
(5)Here, 
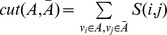
. 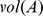 is the volume of the set A. 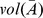 is the volume of the set 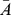. The Ncut spectral method has the strong advantage of being less sensitive to outliers than other graph clustering methods. It has been used in Resting-State fMRI networks [41]. We compared the clustering results of trials to the actual rat behavioral types and computed the correctness of unsupervised partitioning. If the label of a trial was the same as the rat's actual type, the clustering result was considered correct, otherwise, the clustering result was considered to be in error.

### Predicting the behavioral outcomes of single trials

**Figure 5:**
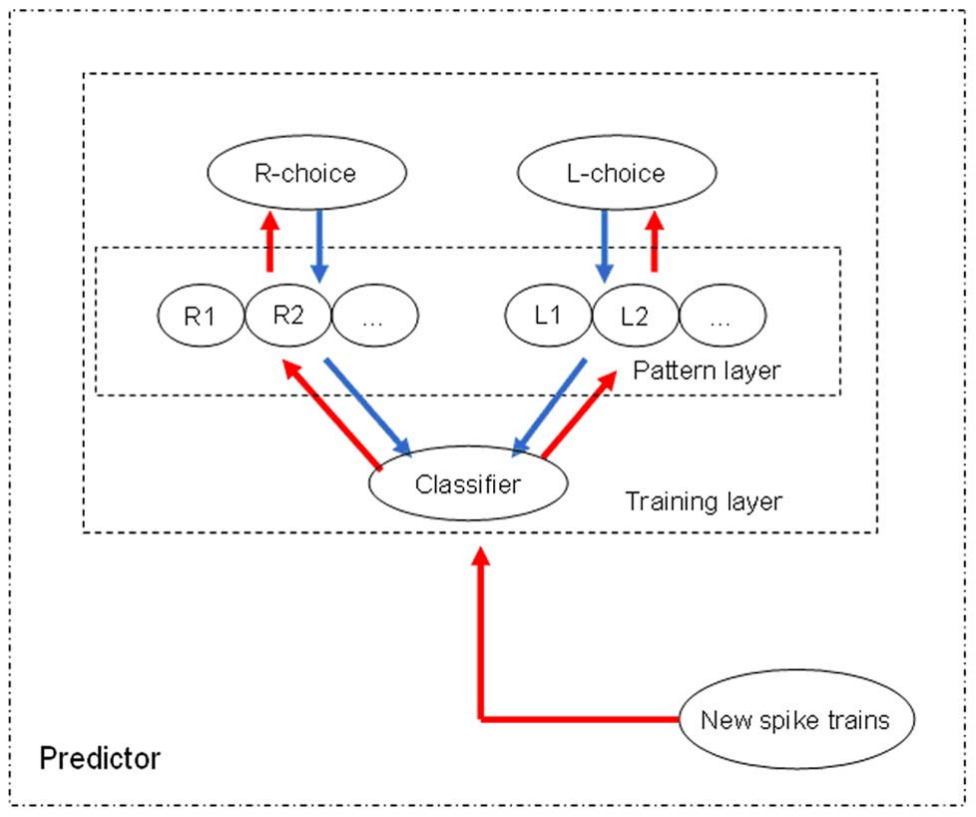
Implementation of the predictive classifier proposed in this paper. A number of trials containing R-choices and L-choices will be trained into a two-cluster classifier. If there is a new trial which we do not know its trial type in advance, connection patterns of this trial will be measured in the pattern layer to decide its trial type.

Unsupervised clustering methods can be used to divide multiple trials of each dataset into two groups according to their self-similarity, corresponding to two different kinds of trial types. It remains unknown, however, whether these neuronal functional networks can be employed to predict the behavioral choices in a single trial. The prediction realization procedure can serve as a classifier for the final prediction stage of the methodology, as shown in Figure 5. The next issue was to determine whether the prediction outcomes of single trials were correct or incorrect. If the predicted result was the same as the rat's actual choice, the prediction was considered correct, otherwise, the prediction was considered to be in error.

We used the leave one cross-validation method (LOO) to evaluate the accuracy of the predictions made using the proposed classifier. Each time that the cross-validation method used a trial as a test data, the remaining N-1 trials served as the training set and trained into a two-cluster classification. This process was repeated N times. Prediction accuracy was here defined as the number of correctly classified trials over the total number of trials. The average accuracy of classification determines the overall accuracy of the prediction classifier. We also realized two methods: pattern matching prediction method and KNN prediction method.

The process of predicting pattern recognition is computed using the following steps: The first step is to select the *i*th trial (1≤*i*≤N). Then the other N-1 trials were classified into two groups (N_1_, N_2_, N_1_+N_2_ = N–1 (N_1_ and N_2_ are numbers of trials in group 1 and 2, respectively) depending on the rat's actual choices. The similarity of *i*th trial to each group was determined by averaging its similarities to all functional connectivity patterns in that group.
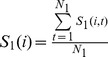
(6)as the average similarities of *i*th trials to all N_1_ trials of group 1.
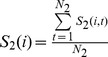
(7)as the average similarities of ith trial to all N2 trials of group 2. In the matching section, the ith trial belonged to the group with maximum similarity argmax j s j (i).

Another method of classification is k-nearest neighbor (KNN) method. The basic idea underlying this algorithm is, for the *i*th trial, selecting the k neighbor trials which have the maximum similarity to the *i*th trial. 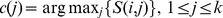 If the number of trials of *c(j)* belonging to group 1 is greater than the number of trials belonging to the group 2, then the label of *i*th trial is assigned to group 1. Otherwise, it is assigned to group 2.

### Performance Evaluation

In order to calculate the correctness of unsupervised clustering of all trials into two classes and the accuracy of predictions of trial outcomes made using the classifier, we used the Jaccard coefficient measure to evaluate the experimental results. The Jaccard coefficient is an intuitive and effective performance evaluation method. In our experiments, unsupervised clustering and prediction classification were all two-way classification problems. Given an initial partition, let 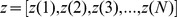 denote the label vector for N trials, 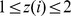. The probability of *i*th trial *v_i_* being classified as group 1 is given by the ratio of the number of the correctly classified trials in group 1 to the number of all trials.
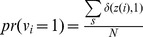
(8)


The entire Jaccard coefficient is defined by the sum of the coefficients of label 1 and 2, respectively.
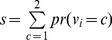
(9)


The value of s is between 0 and 1. The larger the value of s, the better the performance of the experimental results.

### Statistical Tests

One-way ANOVA statistical test was used for statistical comparisons. A p value <0.001 was used as the criterion for a significant statistical difference. A linear regression analysis was conducted to compare the relationship between results of unsupervised clustering and results of single-trial predictions.

## Results

### Unsupervised clustering results

We applied the NJW algorithm and the Ncut algorithm to the ten datasets (using 1 s bins). Gaussian scale parameter was set as σ = 4.

### Average clustering accuracy of Y-maze vs. U-maze

**Figure 6:**
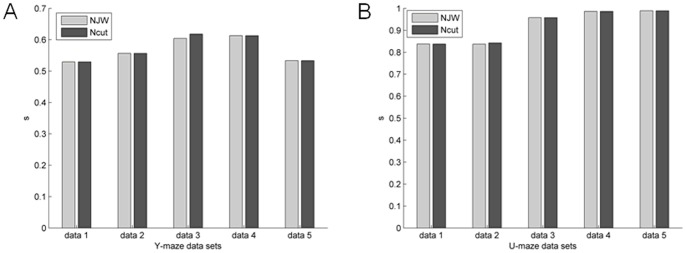
Unsupervised clustering partitioning accuracy of Y-maze and U-maze datasets in the WNFNs using NJW and Ncut. (A) Results of Y-maze datasets. (B) Results of U-maze datasets.

**Figure 7:**
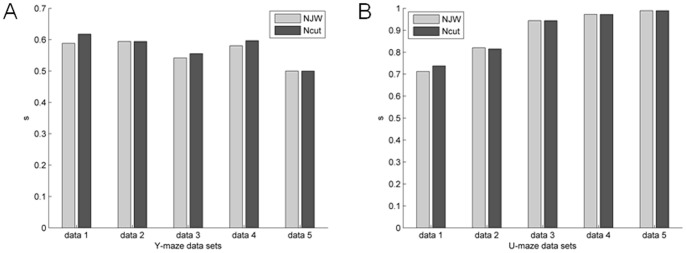
Unsupervised clustering partitioning accuracy of Y-maze and U-maze datasets in the LNCGs using NJW and Ncut. (A) Results of Y-maze datasets. (B) Results of U-maze datasets.

**Figure 8:**
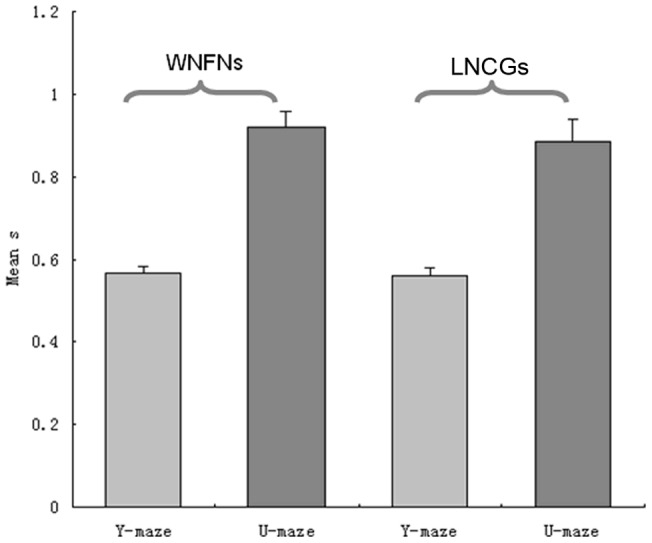
Error bar of partitioning results of ten datasets in the WNFNs, and LNCGs conditions using the NJW algorithm. Error bar represents the standard error.

Figures 6 and 7 are unsupervised two-way clustering results in different network connection structures for Y-maze and the U-maze datasets. The NJW algorithm and Ncut algorithm showed similar results. As shown in Figure 8, results based on the U-maze task were significantly better than those based on the Y-maze task (one-way ANOVA, p<0.001). However, the accuracy of the Y-maze datasets is generally between 50% and 60%.

### Single-trial prediction results

We applied the pattern matching method and KNN method to predictions of the behavioral outcome. The parameter of the number of k nearest neighbor was set as k = 7.

### Average accuracy of predictions of Y-maze vs. U-maze

**Figure 9:**
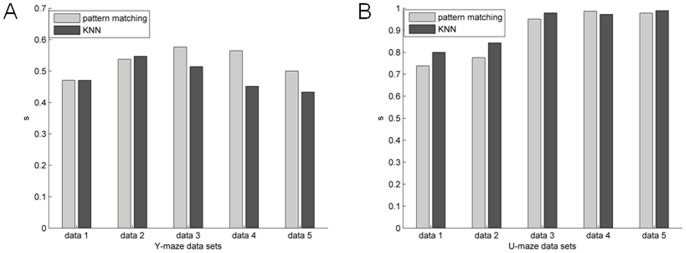
Predictions of trial outcomes in the WNFNs. (A) Results of Y-maze datasets. (B) Results of U-maze datasets.

**Figure 10:**
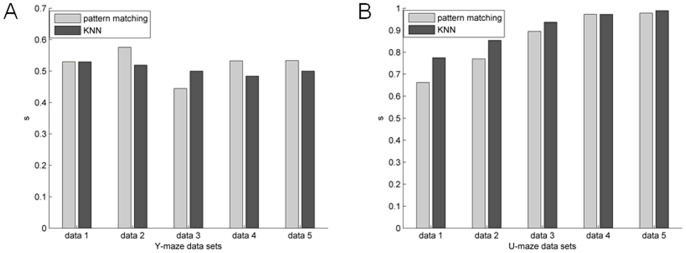
Predictions of trial outcomes in the LNCGs. (A) Results of Y-maze datasets. (B) Results of U-maze datasets.

**Figure 11:**
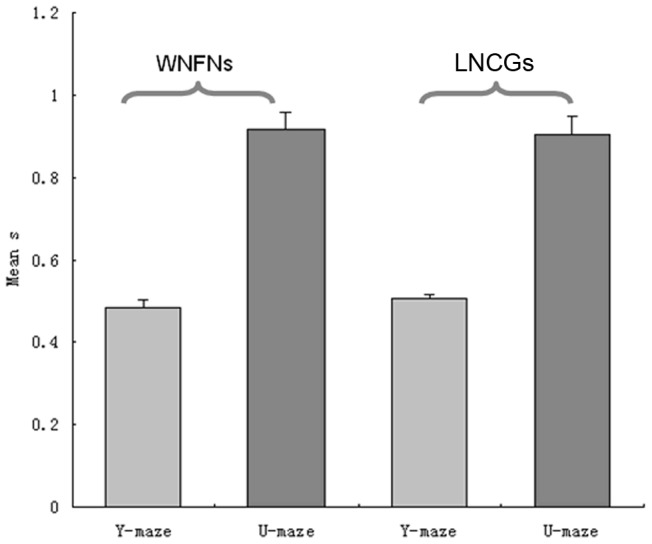
Predictions of trial outcome were based on WNFNs and LNCGs using the KNN prediction method. Error bar represents the standard error.

Figures 9 and 10 show the prediction accuracy of the datasets from two different tasks, respectively. As shown in Figure 11, the accuracy of the prediction Y-maze data sets is low. The value of the Jaccard coefficient was between 0.4 and 0.6, showing that the prediction accuracy rate was generally at about 50%, which is similar to the results generated by chance (one-way ANOVA, p = 0.653). The results of U-maze datasets were higher. The value of the Jaccard coefficient for U-maze datasets was between 0.7 and 1. Trial outcomes were predicted significantly above chance due to the occurrence of different functional connections in two trial types (one-way ANOVA, p<0.001). The results showed that when the rats performed the U-maze task, we were able to more easily take advantage of the functional connectivity among neurons to predict behavior outcomes. For U-maze datasets, the methods proposed in this paper could not only be used to partition two different behaviors effectively, but were also able to predict whether the outcomes of single trials were correct or incorrect.

### Results of unsupervised clustering vs. results of single-trial predictions

To indicate whether certain functional connections were associated with particular behavioral choices, we gave out the relationship of Ncut results to KNN prediction accuracy of Y-maze and U-maze datasets.

**Figure 12:**
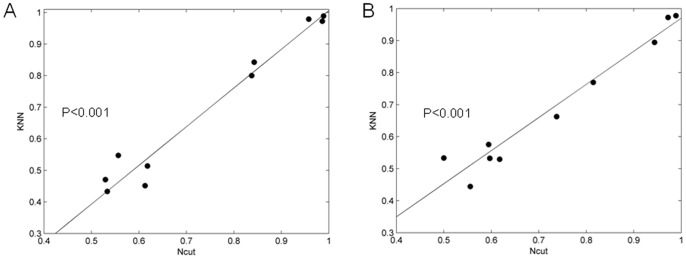
Relationship of Ncut results to KNN results. (A) Results in WNFNs of ten datasets. (B) Results in LNCGs of ten datasets.

As shown in Figure 12, a linear regression analysis indicated that Ncut unsupervised classification accuracy had a strong correlation with KNN prediction accuracy. If we need to determine whether a recorded spike trains dataset can be used to predict behavioral outcomes of rats, the dataset must exhibit obvious differences in functional connectivity between two different kinds of behaviors, and it must have a higher correct rate when it is divided using unsupervised clustering.

### WNFNs vs. LNCGs

**Figure 13:**
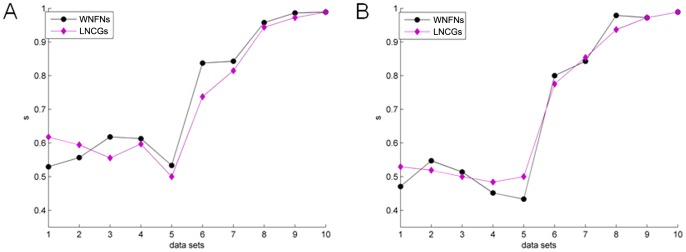
Unsupervised clustering trials and predictions of trial outcomes based on WNFNs and LNCGs. (A) Ncut results. (B) KNN results.

Figure 13 shows a comparison of Ncut and KNN in WNFNs and LNCGs. Results indicate that the two kinds of network conditions did not differ significantly with respect to accuracy. In the present study, the number of recorded neurons is very limited. The performance did not show significant differences when the neuronal functional networks were divided into sub-networks (one-way ANOVA, Ncut, p = 0.8694; KNN, p = 0.9542).

## Discussion

A large number of studies have shown that there are precise time relationships between neuronal firings in cerebral cortex correspondence to behavior [42]. Functional connectivity can be inferred from these neuronal spike trains [43]. The issue of how to use the structure of neuronal networks to predict the functions of the brain has plagued neuroscientists for a long time [44], [45]. Using the functional connections among neurons to predict the functional behavior of the animal is another serious matter. In this study, we present a new method, based on graph theory technology, for analyzing task-related functional networks from multi-electrode recordings. The results show that the presence of certain especially functional structures in cortical neurons is associated with subsequent behavior.

Our study expanded upon earlier works, which used the spatiotemporal activity patterns of rat cortical neurons to predict rat behavior. In our study, the analysis is based on functional connections between pairs of simultaneously recorded neurons on single trials. These correlations are then used to build whole-recorded neuronal functional networks. These networks are then used to divide into local neuronal circuit groups for each trial based on the maximization of modularity Q. To our knowledge, there are no published reports in which the functional connections of neurons were used to study rat behavioral choices.

We found that datasets of U-maze task to have a significant difference from the Y-maze task with respect to functional connections in the two different trial types. The accuracy of unsupervised clustering trials and prediction outcomes of trials was found to be very high, as indicated using different forms of analysis, indicating obvious differences in neuronal functional connections when rats perform clockwise and counterclockwise tasks. However, the accuracy of the Y-maze datasets is low, indicating that there are no particularly obvious differences among the neuronal functional connections associated with the L-choice task and R-choice task.

In the U-maze task, the two different trajectories (from point A to B or from point B to A) are represented much like two different locations would be. This may be related to the place cells within the hippocampus [46]. These cells were found to be sensitive to direction and cells fire at certain location fired when the rat went through the location in a specific direction but not when the rat went through in the opposite direction. The sequential firing patterns may be different at particular locations along the rat's trajectory [47]. It is well established that the spatial representation made by hippocampal neurons on a track differs depending on the running direction of the rat [48], [49]. This may lead to the changes in neuronal functional connections. Our study is consistent with the results of these neuroscience studies. Due to the U-maze task is a simple task and there are place cells in the hippocampus, whether the successful performance of the U-maze task is due to the brain region being recorded or to the task performed by the animal is still not known. These two possibilities all may be existed.

In our experiments, the number of recorded neurons was limited. The constructed neuronal functional networks were relatively simple, and the corresponding generated neuronal circuit groups were also relatively simple. The results of analysis of two functional connectivity cases showed them to have no significant differences. However, the method proposed in this paper still provides meaningful information that may be suitable for future analysis of large-scale neuronal networks. As the number of recorded neurons increased, the partitioning of neuronal networks into circuits became more complex [12], [13]. More complex methods of analysis methods are urgently needed. The greater number of neurons recorded, the more clearly the relationships between the neuronal functional networks and behavior could be revealed.

Although the results of studies that use electrodes to record neuronal activity cannot be translated directly to the human brain functional network, the method proposed in this paper can also be used to analyze the functional community of the brain and investigate human brain disease pathology based on EEG and fMRI [50], [51].

In the present study, the experimental results were controlled by the three parameters, the size of the time window of the bins, the value of the nearest neighbor k, and Gaussian scale parameter σ. In this way, the structures of the neuronal functional networks differed as the size parameters differed. These parameters were found to directly affect the results of the experiment, and we constructed a number of networks using different parameters. Although some variations appeared in the results, these did not contradict the conclusions of this paper, so no details of these results are provided here. Parameter selection was the most difficult part of the study, and most decisions were based on trial and error. The best way to improve this study would be to reduce the use of parameters.

The current study was limited by the multi-electrode recordings and the method of online spike sorting. In our experiments, although the neuronal activity was observed in vivo, individual neurons were sorted offline by hand, which was highly subjective. In this way, it also limits the ability to predict animal behavior by using online firing patterns of neurons. In addition, a community structure partitioning method based on the maximization of modularity Q has the limitations as a “resolution limit” [52], [53].

In conclusion, using the activity patterns of cortical neurons to predict animal behavior of the animals is a key issue in neuroscience. The achievements made in this area may help to solve the brain machine interface problems and facilitate the development of techniques that could promote the recovery of behavioral function after brain disease. This paper presents a new method that incorporates the neuronal functional networks to predict the trial types of rats. We performed an experimental comparison of two kinds of cognitive behavioral tasks datasets. The results show that this method can predict the behavior of different rats effectively. The results presented here provide definite evidence that neuronal functional networks exist in the cortex of moving rats of a behavioral task and that the different networks become active during different tasks. We believe that these results, along with the progress that has recently been made in neuronal recording techniques and in prediction methods, may improve the researchers' ability to predict animal behavior through the functional networks.

## References

[1] Pesaran B, Pezaris JS, Sahani M, Mitra, Andersen RA (2002) Temporal structure in neuronal activity during working memory in macaque parietal cortex. Nat Neurosci 5: 805–811. 12134152 10.1038/nn890

[2] Quiroga RQ, Panzeri S (2009) Extracting information from neuronal populations: information theory and decoding approaches. Nature Rev Neurosci 10: 173–185. 19229240 10.1038/nrn2578

[3] Fellous JM, Tiesinga PH, Thomas PJ, Sejnowski TJ (2004) Discovering spike patterns in neuronal responses. J Neurosci 24: 2989–3001. 15044538 10.1523/JNEUROSCI.4649-03.2004 PMC2928855

[4] Ikegaya Y, Aaron G, Cossart R, Aronov D, Lampl I, et al (2004) Synfire chains and cortical songs: Temporal modules of cortical activity. Science 304: 559–564. 15105494 10.1126/science.1093173

[5] Talwar SK, Xu S, Hawley ES, Weiss SA, Moxon KA, et al (2002) Rat navigation guided by remote control. Nature 417: 37–38. 11986657 10.1038/417037a

[6] Villa AEP, Tetko IV, Hyland B, Najem A (1999) Spatiotemporal activity patterns of rat cortical neurons predict responses in a conditioned task. Proceedings of the National Academy of Sciences 96(3): 1106–1111. 10.1073/pnas.96.3.1106 PMC15358 9927701

[7] Laubach M, Wessberg J, Nicolelis MAL (2000) Cortical ensemble activity increasingly predicts behaviour outcomes during learning of a motor task. Nature 405: 567–571. 10850715 10.1038/35014604

[8] Ress D, Backus BT, Heeger DJ (2000) Activity in primary visual cortex predicts performance in a visual detection task. Nat Neurosci 3: 940–945. 10966626 10.1038/78856

[9] Wessberg J, Stambaugh CR, Kralik JD, Beck PD, Laubach M, et al (2000) Real-time prediction of hand trajectory by ensembles of cortical neurons in primates. Nature 408: 361–365. 11099043 10.1038/35042582

[10] Chapin JK (2004) Using multi-neuron population recordings for neural prosthetics. Nat Neurosci 7: 452–455. 15114357 10.1038/nn1234

[11] Chapin JK, Moxon KA, Markowitz RS, Nicolelis MAL (1999) Real-time control of a robot arm using simultaneously recorded neurons in the motor cortex. Nat Neurosci 2: 664–670. 10404201 10.1038/10223

[12] Stevenson IH, Kording KP (2011) How advances in neural recording affect data analysis. Nat Neurosci 14: 139–142. 21270781 10.1038/nn.2731 PMC3410539

[13] Lin L, Chen G, Xie K, Zaia KA, Zhang S, et al (2006) Large-scale neural ensemble recording in the brains of freely behaving mice. J Neurosci Meth 155: 28–38. 10.1016/j.jneumeth.2005.12.032 16554093

[14] Wilson MA, McNaughton BL (1994) Reactivation of hippocampal ensemble memories during sleep. Science 265: 676–679. 8036517 10.1126/science.8036517

[15] Brown EN, Kass RE (2004) Mitra (2004) Multiple neural spike train data analysis: state-of-the-art and future challenges. Nat Neurosci 7: 456–461. 15114358 10.1038/nn1228

[16] Behrens TE, Sporns O (2012) Human connectomics. Curr. Opin. Neurobiol. 22: 144–153. 10.1016/j.conb.2011.08.005 PMC3294015 21908183

[17] Sporns O, Tononi G, Kotter R (2005) The human connectome: A structural description of the human brain. PLoS Comput Biol 1: e42. 16201007 10.1371/journal.pcbi.0010042 PMC1239902

[18] Bullmore E, Sporns O (2009) Complex brain networks: graph theoretical analysis of structural and functional systems. Nature Reviews Neuroscience 10: 186–198. 19190637 10.1038/nrn2575

[19] Kaiser M (2011) A tutorial in connectome analysis: Topological and spatial features of brain networks. Neuroimage 57: 892–907. 21605688 10.1016/j.neuroimage.2011.05.025

[20] Sporns O, Zwi JD (2004) The small world of the cerebral cortex. Neuroinformatics 2: 145–162. 15319512 10.1385/NI:2:2:145

[21] Li L, Zhang JX, Jiang T (2011) Visual Working Memory Load-Related Changes in Neural Activity and Functional Connectivity. PLoS ONE 6(7): e22357. 21789253 10.1371/journal.pone.0022357 PMC3138779

[22] Bai F, Shu N, Yuan Y, Shi Y, Yu H, et al (2012) Topologically convergent and divergent structural connectivity patterns between patients with remitted geriatric depression and amnestic mild cognitive impairment. J Neurosci 32: 4307–4318. 22442092 10.1523/JNEUROSCI.5061-11.2012 PMC6621223

[23] Sporns O, Honey CJ, Kötter R, (2007) Identification and classification of hubs in brain networks. PLoS ONE 2, e1049. 10.1371/journal.pone.0001049 PMC2013941 17940613

[24] Wang J, Zuo X, He Y (2010) Graph-based network analysis of resting-state functional MRI. Front Syst Neurosci 4: 16. 20589099 10.3389/fnsys.2010.00016 PMC2893007

[25] Achard S, Salvador R, Whitcher B, Suckling J, Bullmore E (2006) A resilient, low-frequency, small-world human brain functional network with highly connected association cortical hubs. J Neurosci 26: 63–72. 16399673 10.1523/JNEUROSCI.3874-05.2006 PMC6674299

[26] Gerhard F, Pipa G, Lima B, Neuenschwander S, Gerstner W (2011) Extraction of network topology from multi-electrode recordings: is there a small-world effect? Front Comput Neurosci 5: doi: 10.3389/fncom.2011.00004 10.3389/fncom.2011.00004 PMC3036953 21344015

[27] Schreiber S, Fellous JM, Whitmer D, Tiesinga P, Sejnowski TJ (2003) A new correlation-based measure of spike timing reliability. Neurocomputing 52: 925–931. 20740049 10.1016/S0925-2312(02)00838-X PMC2926980

[28] Lopes-dos-Santos V, Conde-Ocazionez S, Nicolelis MAL, Ribeiro ST, Tort ABL (2011) Neuronal Assembly Detection and Cell Membership Specification by Principal Component Analysis. PLoS ONE 6(6): e20996. 21698248 10.1371/journal.pone.0020996 PMC3115970

[29] He Y, Wang J, Wang L, Chen ZJ, Yan C, et al (2009) Uncovering Intrinsic Modular Organization of Spontaneous Brain Activity in Humans. PLoS ONE 4(4): e5226. 19381298 10.1371/journal.pone.0005226 PMC2668183

[30] Paiva A, Park I, Principe J (2009) A comparison of binless spike train measures. Neural Computing and Applications 18: 1–22.

[31] Ahmadlou M, Adeli H (2011) Fuzzy synchronization likelihood for automated EEG-based diagnosis of attention-deficit/hyperactivity disorder. Clinical EEG and Neuroscience 42: 6–13. 21309437 10.1177/155005941104200105

[32] Liang Z, King J, Zhang N (2011) Uncovering intrinsic connectional architecture of functional networks in awake rat brain. The Journal of neuroscience: the official journal of the Society for Neuroscience 31: 3776–3783. 21389232 10.1523/JNEUROSCI.4557-10.2011 PMC3073070

[33] Newman ME (2006) Finding community structure in networks using the eigenvectors of matrices. Phys. Rev. E 74, 036104. 10.1103/PhysRevE.74.036104 17025705

[34] Rubinov M, Sporns O (2010) Complex network measures of brain connectivity: Uses and interpretations. Neuroimage 52: 1059–1069. 19819337 10.1016/j.neuroimage.2009.10.003

[35] Newman ME (2006) Modularity and community structure in networks. Proc Natl Acad Sci U S A 103: 8577–8582. 16723398 10.1073/pnas.0601602103 PMC1482622

[36] Schwarz AJ, Gozzi A, Bifone A (2009) Community structure in networks of functional connectivity: resolving functional organization in the rat brain with pharmacological MRI. Neuroimage 47: 302–311. 19345737 10.1016/j.neuroimage.2009.03.064

[37] Shen X, Papademetris X, Constable RT (2010) Graph-theory based parcellation of functional subunits in the brain from resting-state fMRI data. Neuroimage 50: 1027–1035. 20060479 10.1016/j.neuroimage.2009.12.119 PMC3062848

[38] Meunier D, Achard S, Morcom A, Bullmore E (2009) Age-related changes in modular organization of human brain functional networks. Neuroimage 44: 715–723. 19027073 10.1016/j.neuroimage.2008.09.062

[39] Ng AY, Jordan MI, Weiss Y (2001) On spectral clustering: analysis and an algorithm. Adv. in Neural Inform. Proces. Syst. (NIPS) 849–856.

[40] Shi J, Malik J (2000) Normalized cuts and image segmentation. IEEE Trans. Pattern Anal. Machine Intell. 22: 888–905.

[41] van den Heuvel M, Mandl R, Hulshoff Pol H (2008) Normalized Cut Group Clustering of Resting-State fMRI Data. PLoS ONE 3(4): e2001. 18431486 10.1371/journal.pone.0002001 PMC2291558

[42] Shmiel T, Drori R, Shmiel O, BenShaul Y, Nadasdy Z, et al (2005) Neurons of the cerebral cortex exhibit precise interspike timing in correspondence to behavior. Proc. Natl. Acad. Sci. U.S.A. 102: 18655–18657. 10.1073/pnas.0509346102 PMC1317975 16339894

[43] Feldt S, Bonifazi P, Cossart R (2011) Dissecting functional connectivity of neuronal microcircuits: experimental and theoretical insights. Trends Neurosci 34: 225–236. 21459463 10.1016/j.tins.2011.02.007

[44] Honey CJ, Thivierge JP, Sporns O (2010) Can structure predict function in the human brain? NeuroImage 52: 766–776. 20116438 10.1016/j.neuroimage.2010.01.071

[45] Honey CJ, Sporns O, Cammoun L, Gigandet X, Thiran JP, et al (2009) Predicting human resting-state functional connectivity from structural connectivity. Proc Natl Acad Sci. U. S. A. 106: 2035–2040. 10.1073/pnas.0811168106 PMC2634800 19188601

[46] O'Keefe J, Dostrovsky J (1971) The hippocampus as a spatial map: Preliminary evidence from unit activity in the freely-moving rat. Brain research 34: 171–175. 5124915 10.1016/0006-8993(71)90358-1

[47] Fenton AA, Muller RU (1998) Place cell discharge is extremely variable during individual passes of the rat through the firing field. Proc Natl Acad Sci. U. S. A (95) 3182–3187. 10.1073/pnas.95.6.3182 PMC19716 9501237

[48] McNaughton BL, Barnes CA, O'Keefe J (1983) The contributions of position, direction, and velocity to single unit activity in the hippocampus of freely-moving rats. Experimental Brain Research 52: 41–49. 6628596 10.1007/BF00237147

[49] O'Keefe J (1993) Recce (1993) Phase relationship between hippocampal place units and the EEG theta rhythm. Hippocampus 3: 317–330. 8353611 10.1002/hipo.450030307

[50] Ahmadlou M, Adeli H (2011) Functional community analysis of brain: a new approach for EEG-based investigation of the brain pathology. Neuroimage 58: 401–408. 21586331 10.1016/j.neuroimage.2011.04.070

[51] Laurienti P, Hugenschmidt CE, Hayasaka S (2009) Modularity maps reveal community structure in the resting human brain. Nature Precedings. Available: http://hdl.handle.net/10101/npre.2009.3069.1.

[52] Lu H, Wei H (2012) Detection of community structure in networks based on community coefficients. physica A 391: 6156–6164.

[53] Fortunato S, Barthelemy M (2007) Resolution limit in community detection. Proc. Natl. Acad. Sci. U. S. A. 104: 36–41. 10.1073/pnas.0605965104 PMC1765466 17190818

